# A Small-Scale Object Detection Algorithm in Intelligent Transportation Scenarios

**DOI:** 10.3390/e26110920

**Published:** 2024-10-29

**Authors:** Junzi Song, Chunyan Han, Chenni Wu

**Affiliations:** School of Software, Northeastern University (NEU), Shenyang 110169, China

**Keywords:** intelligent transportation, small object detection, YOLOv4 tiny, feature pyramid, information entropy

## Abstract

In response to the problem of poor detection ability of object detection models for small-scale targets in intelligent transportation scenarios, a fusion method is proposed to enhance the features of small-scale targets, starting from feature utilization and fusion methods. The algorithm is based on the YOLOv4 tiny framework and enhances the utilization of shallow and mid-level features on the basis of Feature Pyramid Network (FPN), improving the detection accuracy of small and medium-sized targets. In view of the problem that the background of the intelligent traffic scene image is cluttered, and there is more redundant information, the Convolutional Block Attention Module (CBAM) is used to improve the attention of the model to the traffic target. To address the problem of data imbalance and prior bounding box adaptation in custom traffic data sets that expand traffic images in COCO and VOC, we propose a Copy-Paste method with an improved generation method and a K-means algorithm with improved distance measurement to enhance the model’s detection ability for corresponding categories. Comparative experiments were conducted on a customized 260-thousand traffic data set containing public traffic images, and the results showed that compared to YOLOv4 tiny, the proposed algorithm improved mAP by 4.9% while still ensuring the real-time performance of the model.

## 1. Introduction

For object detection models applied to intelligent transportation, not only should accuracy be emphasized, but also the speed of model detection, requiring a balance between accuracy and speed. In the intelligent traffic scenario, vehicles, pedestrians, and other targets tend to have smaller scales. Especially when the vehicle is traveling too fast, if these targets cannot be detected in time and accurately, it will have a serious impact on the accurate operation of the subsequent intelligent traffic system. In recent years, although the overall detection performance of object detection has been greatly improved, the research progress of small object detection is relatively slow, and models in intelligent transportation scenarios require real-time performance. Therefore, further exploration is still needed for small object detection methods in intelligent transportation.

The limitation of small object detection capability is partly due to the imbalance in target scale in training data and partly due to the limitations of the detection network itself [1]. For most data sets, medium-to-large-scale targets account for the majority, while small-scale targets only account for a small proportion. For the model, good detection of medium- and large-scale targets will bring more gains, so the detection of small-scale targets will be ignored. For the structural part of the model itself, in order to obtain more deep-seated semantic information, most detection networks use more convolutional and pooling layers to stack, and multi-layer stacking will cause the information of small targets to gradually disappear as the network layer propagates [2], resulting in the inability to detect small targets well. The Feature Pyramid Network (FPN) [3] proposed by Lin T.Y. et al. and the Path Aggregation Network (PAN) [4] used in YOLOv4 alleviate the problem of information loss to some extent by fusing shallow and deep feature maps. However, their utilization and fusion of shallow and deep information, as well as their complexity, still need further improvement. On the basis of FPN and PAN, a group of feature utilization and fusion methods with more complex structures has emerged. The common problem is that improving accuracy increases model complexity, which can affect the running speed of the model.

Based on the above analysis, considering that the object detection model in intelligent transportation scenes needs to ensure real-time performance, the main methods to solve the problem of small object detection include data processing and multi-scale feature fusion [5]. This article mainly improves the data processing and detection model structure to improve the object detection effect in intelligent transportation scenes. In terms of model structure, for the detection of small-scale targets, the feature fusion method is enhanced on the basis of FPN to enhance the model’s detection ability. The attention mechanism Convolutional Block Attention Module (CBAM) is also used to further enhance detection accuracy, while ensuring that the model still has real-time performance after the above improvements. In terms of data processing, to address the problem of imbalanced data sets with small samples and targets, an improved Copy-Paste method is used for corresponding feature enhancement, effectively enhancing the model’s detection ability for these targets. Subsequently, in response to the adaptation problem between the model’s prior bounding boxes and the traffic data set, an improved K-means algorithm is used for prior bounding box clustering to obtain prior bounding boxes that fit the custom traffic data set and improve the model’s detection accuracy for each category.

Finally, we designed a series of experiments to prove our conclusion using a customized 300,000 traffic data set as the training and testing set. The improved model based on the PF Net feature fusion structure proposed in this article increased mAP by 2.01%. After adding three CBAM modules, the mAP of the model increased by 4.03%. For small targets of concern, taking the reflector cone as an example, the final improved model PF-YOLOv4 tiny CBAM can increase by 1.69 percentage points. After using the improved Copy-Paste data augmentation method for small-scale targets, the detection accuracy has improved by at least 1%; On the basis of the above, K-means [6] was used for prior bounding box clustering, which improved the detection accuracy of some categories by 3%.

In summary, our main contributions are as follows:

- We propose an improved feature fusion structure Point Fractal Network (PF Net) based on FPN, which ensures real-time performance while further improving accuracy.
- An improved model PF-YOLOv4 tiny CBAM with an added CBAM attention module has been proposed, which makes the model pay more attention to the targets in the image, further improves the detection accuracy of the model, and ensures that the improved model can meet the real-time requirements of intelligent transportation scenarios.
- A data augmentation method based on Copy-Paste improvement has been proposed to enhance the detection ability of small targets in custom traffic data sets.
- A K-means method for improving distance measurement has been proposed, which can be applied to custom traffic data sets to obtain more suitable prior bounding box and further improve the detection performance of targets.

## 2. Related Work

### 2.1. Object Detection Model Based on Deep Learning

With the development of deep learning, a large number of object detection models based on deep learning have been proposed. Object detection based on deep learning mainly obtains the object category and object position in the image through convolutional network. Generally, it can be divided into a one-stage model and a two-stage model. The first model that used deep learning to solve the object detection problem was the Region-based Convolutional Neural Network (R-CNN) [7], which firstly obtained the candidate region through the traditional extraction method and then obtained the object category and location through the convolutional network. Compared with other traditional motion modeling object detection methods and machine learning object detection methods, it was a great breakthrough, with great performance improvement. After that, the Spatial Pyramid Pooling Network (SPP-Net) [8] continued to use the convolutional structure of deep learning and innovatively proposed the space pyramid pool. Later, Ross Girshick proposed the Fast R-CNN network [9] and introduced Region of Interest (ROI) Pooling of areas of interest. ROI Pooling optimized the problem of R-CNN repeatedly extracting multiple candidate areas in an image and improved the efficiency of image processing. On the basis of the Fast R-CNN network, Faster R-CNN [10] was proposed, which used the Regional Proposal Network (RPN) structure to generate candidate regions and eliminated the traditional selective search, thus improving the execution efficiency. At the same time, the concept of anchor box was proposed for the first time in Faster R-CNN to realize end-to-end object detection and further improve the precision of small object detection. At the same time, Faster R-CNN had a great improvement in speed compared with the previous models. All of the above models belong to the two-stage object detection model. The two-stage model first extracts the candidate region, then uses the convolutional network to obtain the approximate position and final position of the target and other relevant information.

In the field of intelligent transportation, although the two-stage model has high accuracy, its running speed is limited, and it cannot meet the requirements of real-time performance. Therefore, the one-stage object detection model without additional extraction of candidate regions was proposed, which only requires a convolutional network to obtain the location and category of the object. The representative models of the single-stage object detection model include the YOLO series model and the Single Shot MultiBox Detector (SSD), etc. Based on the YOLO model, Liu W et al. proposed the SSD [11] model. The SSD model followed the concept of the anchor box. The CNN structure was used for direct detection during detection, and the multi-scale feature map was not only used for the feature map of the last convolutional layer. Multiscale detection of SSD improved the detection ability of the model for small targets. Subsequently, YOLOv3 [12], a very representative model in the YOLO series, adopted the structure of feature pyramid and three detection heads, corresponding to the detection of large, medium and small targets, significantly improving the detection ability of the model for small targets. In 2020, a paper proposing YOLOv4 was published. YOLOv4 combined a variety of new techniques in the field of deep learning, especially Mosaic, a self-antagonistic training data enhancement method, and the innovative feature fusion structure of PAN. These data enhancement methods amplified the target features, and the PAN structure further improved the feature fusion method. The shallow and deep information fusion was strengthened to improve the detection effect of targets at various scales. YOLOv4 papers also published a variety of models of different complexity, such as YOLOv4 tiny. The structure of YOLOv4 tiny was more simplified, and the relatively simple FPN structure was used to facilitate the selection of appropriate models according to the required precision and real-time requirements. The strategies and techniques used in the later YOLOv5 and YOLOv4 are roughly the same. More engineering optimization logic was introduced, and a variety of lightweight models with different complexities were designed, such as YOLOv5s. The above model is an anchor-based object detection model. With its development, anchor-free object detection models have been proposed continuously in recent years. For example, CornerNet (Detecting Objects as Paired Keypoints) [13] and Feature Selective Anchor-Free (FSAF) Module for Single-Shot Object Detection [14] are the first work of anchor free, providing new ideas for object detection while avoiding some disadvantages brought by the anchor mechanism. It is another branch of the object detection one-stage model.

The performance of the above representative one-stage and two-stage object detection models was comprehensively compared. Currently, the one-stage model mentioned above is still the main model used for object detection in the intelligent transportation scene. At the same time, although the object detection model has been constantly developed and replaced, and even new detection ideas have appeared, there has been no great breakthrough in small object detection. This paper takes YOLOv4 tiny, which is widely used in industry, as the benchmark model. YOLOv4 tiny is a lightweight model that can fully guarantee real-time performance, so it is improved based on it.

### 2.2. Data-Based

Data-based methods solve problems from the data set itself, and such methods tend to be effective only for specific data sets, such as COCO. There are two imbalance problems in COCO data sets: image-level and instance-level imbalance. Image-level imbalance means that only 51.8% of pictures contain small objects in COCO. The representative method to solve this problem is Stitcher. Stitcher [15] takes the proportion of small object loss in the total loss as the feedback signal. When the proportion is less than a certain threshold, the four pictures will be combined into one picture as the input of the next iteration, which is equivalent to increasing the number of small objects. Instance-level imbalance means that the pixel area of small- and medium-sized objects occupies 1% in COCO, so data enhancement is usually adopted. The data enhancement method can specifically improve the number of features of the specified target, not only enhancing the generalization of the model but also balancing the data set, so as to improve the detection ability of the corresponding target without affecting the real-time application of the model.

One of the reasons why small targets are difficult to detect is the unbalance of large and small target samples. Generally, medium- and large-scale targets occupy a large proportion in the public data set of general scenes or the data sets of intelligent transportation scenes, which leads to more attention to the detection ability of medium- and large-scale targets in the process of model learning. At the same time, when the artificially set prior bounding box size is significantly different from the real bounding box, it will lead to fewer positive training samples for small targets and more unbalanced anchors matching medium and large targets, thus making the model ignore the detection of small targets. Therefore, data enhancement is widely used in object detection models to improve the detection ability of small targets. YOLOv4 proposed the Mosaic data enhancement model, which used four images to splice a new image sample, enriched the background of the detected target, transformed the large target of the original image into small target, expanded the number of small targets in the data set, and improved the detection ability of the model on small targets. Kisantal M. et al. raised oversampling of small target training samples and copy and paste small targets in the COCO sample of the public data set, using this method to provide enough of the small target and anchor matching, in turn, to promote small object detection ability. It has been proved that the Copy-Paste method is effective in improving small object detection. Reference [16] has proved the effectiveness of Copy-Paste in instance segmentation and the universal validity of the data enhancement method. However, Copy-Paste in the literature [1] copies and pastes back the original image to achieve small-target data enhancement, which can cause an imbalance in the data set to a certain extent. Because the procedure only increases the number of small targets, it does not increase the number of images containing small targets.

Our method uses an improved Copy-Paste idea, which increases the number of small targets and the number of pictures containing small targets at the same time, thus improving the detection ability of the model on small targets. As described in the previous data enhancement section, copying a small target to multiple positions in the picture can increase the number of anchor matched by small targets, increase the training weight of small targets, and reduce the bias of the network to large targets. Similarly, in reverse thinking, if the data set has been determined, the setting strategy of the anchor responsible for small targets can also be added to make the learning of small targets more adequate in the training process. For example, in FaceBoxes [17], one of the contributions is the anchor densification strategy, which enables different types of anchors to have the same density on the image, significantly improving the recall rate of small-scale faces. Single Shot Scale-invariant Face Detector (S3FD) [18] reduces the threshold of Intersection over Union (IoU) for positive samples of small objects and relieves the threshold of IoU to 0.1 for a small number of positive samples. Dot Distance [19] designed a new index DotD to replace IoU for the allocation standard of positive and negative samples. Simply put, IoU was replaced by the distance function of two bounding box centers. Therefore, in the data set after data enhancement, consideration about anchor is further added in this paper. The K-means algorithm is used to conduct scale clustering on the marked targets in the data set to obtain the anchor proportion suitable for the data set, so as to improve the adaptation ability of the model’s prior bounding box to small targets during model training and ensure more adequate model learning.

### 2.3. Multi-Scale Feature Fusion

Another effective way to improve model detection capabilities is to make full use of the multi-scale features generated during the model of model convolution. This method is not only based on the principle of intuitive image pyramids but also the key concepts in information theory, such as information entropy and cross-entropy, through reasonable characteristic coding and decoding processes to improve the accuracy and robustness of target detection.

In information theory, information entropy is an important indicator of the amount of information, indicating the size of random variable uncertainty. In target detection, the amount of information of different scale features contains different amounts of information. The shallow layers of features usually include more details (such as edges and textures), while deep features pay more attention to semantic information (such as object categories). Through the calculation of information entropy, we can quantify the importance of the characteristics of each scale, thereby optimizing the feature selection strategy to improve the effectiveness and efficiency of the model [20].

Cross entropy is used as a common loss function in machine learning, and the difference between the real distribution and the prediction distribution is measured. In the target detection model, designing a cross-entropy-based loss function can effectively train the model to make its output closer to the real target position and category. In addition, for the combination of multi-scale features, the cross-level entropy loss can be used to calculate the loss for the characteristic diagrams of each scale and then weight the summary to guide the model more accurately to learn and use multi-scale features [21].

The characteristic fusion process can be regarded as a process of information coding and decoding. The encoder (convolutional layer) is responsible for extracting multi-level feature information from the input image, and this information encodes in a specific way (such as the form of the feature diagram). The decoder (usually a sampling or feature fusion layer) is responsible for decoding the encoded feature information back to the space domain to form the final detection results. In multi-scale feature fusion, the encoder is responsible for extracting the multi-scale characteristics, while the decoder effectively integrates these features through specific fusion strategies (such as FPN, PAN) to improve the model’s detection capacity.

SSD is the first object detection model that attempts to use multi-scale feature maps for prediction. It performs detection on six feature maps of different scale sizes, which improves the detection ability of the model for multi-scale targets, including small targets. FPN proposed the concept of feature pyramid for the first time and fused the feature information of different downsampling rates to improve the feature expression ability. On the basis of FPN, YOLOv4 improved the structure and designed PAN. PAN added a branch on FPN to obtain better detection effect. In addition, there are many other kinds of feature utilization and fusion methods in the object detection model. For example, the simple bidirectional fusion represents the Bi-directional Feature Pyramid Network (BiFPN) [22], and the BiFPN is an improvement on the PAN bidirectional foundation. Multiple BiFPNs can be used in series, and the BiFPN will increase the amount of computation compared with PAN. In addition to simple bidirectional fusion, there are more complex bidirectional fusions, such as Adaptively Spatial Feature Fusion (ASFF) [23] and Recursive-FPN [24]. Based on FPN, ASFF studies the effects of each stage and further integrates the effects of the three stage features. The integration of different stage features uses the attention mechanism, so that the contribution of other stages to the stage features can be controlled. Recursive-FPN refers to the output of the fusion of traditional FPNS, which is then input to the backbone for a secondary cycle. To sum up, the above methods are to carry out the repeated flow and fusion of characteristic information in different directions to achieve the purpose of improving accuracy. However, such methods will greatly improve the complexity of the model. The above feature fusion methods with better accuracy often have difficulty ensuring the real-time performance of the model, so they cannot be easily used in intelligent transportation scenes. In our method, instead of blindly improving the complexity of the feature fusion structure to improve the model accuracy, we combined FPN and PAN structure to design a real-time PF-Net, which can improve the object detection accuracy, especially the detection accuracy of small targets.

### 2.4. YOLO in Intelligent Transportation

As one-stage algorithms with high real-time performance, Yolo’s various versions have been widely used in the field of intelligent transportation. Liangfu Ge et al. proposed a full bridge traffic load distribution monitoring method using YOLOv3 machine vision technology [25]. This method uses the YOLOv3 algorithm to monitor real-time traffic flow on bridges and accurately identify vehicle types, speeds, and locations, thereby achieving precise evaluation of the distribution of traffic loads on the entire bridge. This method has the characteristics of strong robustness and high accuracy, providing strong support for the health monitoring and safety assessment of bridge structures. At the same time, they also proposed another application of the YOLO algorithm, an improved system for long-term monitoring of traffic load distribution across long-span bridges [26]. This system combines advanced sensor technology and the YOLO algorithm to monitor traffic flow and vehicle loads on bridges in real time and, through data analysis and processing, achieves the long-term monitoring and evaluation of bridge structural status. The improvement of this system lies in the increased accuracy and stability of monitoring, providing strong guarantees for the safe operation of large-span bridges. In addition, Chengjian Liu et al. proposed an intelligent traffic monitoring system based on the YOLO algorithm and a convolutional fuzzy neural network [27]. The system utilizes the YOLO algorithm to perform real-time detection and recognition of vehicles in traffic scenes, while combining convolutional fuzzy neural networks to predict and analyze parameters such as traffic flow and speed. This system has the characteristics of strong real-time performance and high accuracy, which can provide strong support for urban traffic management and planning. Naif Al Mudawi et al. used an updated version of YOLOV8 and a deep belief network to achieve accurate detection and classification of vehicles in aerial image sequences [28]. The YOLOv8 algorithm has faster detection speed and higher accuracy, while deep belief networks can further improve classification accuracy. This study provides effective technical means for fields such as intelligent transportation systems and air traffic monitoring.

## 3. Methods

In this paper, we focus on improving the detection capability of the object detection model for small-scale targets in intelligent transportation scenarios. Our model is improved based on the YOLOv4 tiny structure, which is mainly divided into structural optimization and data-based processing. In terms of data, data enhancement is adopted. The improved Copy-Paste data enhancement method is mainly used to increase the number of samples and features of small targets. At the same time, in order to make the prior bounding box better match with the small target in the custom data set, the K-means clustering method with improved distance measure is used to obtain a more appropriate scale of the prior bounding box. In terms of structure, the original feature pyramid of YOLOv4 tiny is changed, and a new feature utilization and fusion mode between FPN and PAN is proposed, which is named PF-Net. While ensuring real-time performance, the model’s detection ability for multi-scale targets, especially small targets, is improved. Finally, a CBAM attention module is added into the network to improve the model’s attention to the target in the image and further improve the detection ability of small target. In the next few sections, we cover the structure of the model (Section 3.1) and data-based processing (Section 3.2).

### 3.1. Model Structure

This section mainly introduces the improved multi-scale feature fusion of PF-Net based on FPN and the use of a CBAM.

#### 3.1.1. Model Multi-Scale Feature Fusion Method

**Figure 1:**
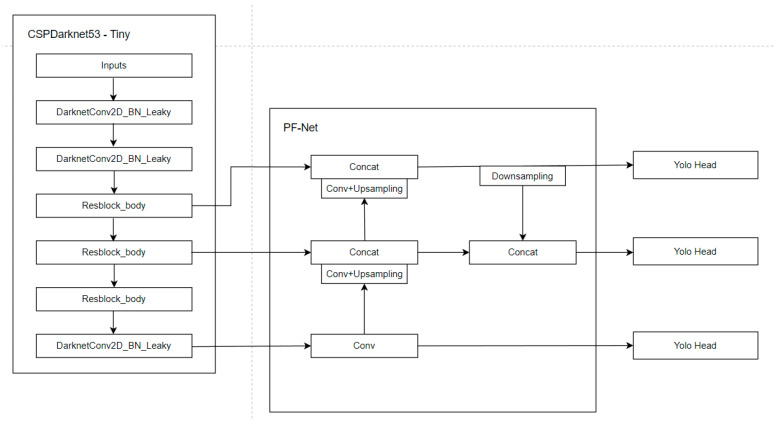
PF-YOLOv4 tiny network structure.

In order to control the complexity of the model to ensure real-time performance and improve the detection accuracy of the model, especially the detection accuracy of small targets, the PF-YOLOv4 tiny model is proposed in this paper. The overall structure and feature utilization and fusion of PF-YOLOv4 tiny are shown in Figure 1.

On the basis of the original two detection heads of YOLOv4 tiny, one detection head is added to the lower sampling layer. Three detection heads can enhance the detection ability of the model to multi-scale targets. Since shallow features are beneficial to small object detection, this operation can enhance the detection ability of the model to small targets. In addition, a new multi-scale feature fusion structure PF-Net is designed, based on the feature utilization and fusion modes of FPN and PANet. On the basis of YOLOv4 tiny, the FPN structure is replaced, and an improved multi-scale feature fusion structure PF-Net is used as the feature utilization and fusion mode of PF-YOLOv4 tiny. PF-YOLOv4 tiny increases the application of network to shallow features, which can improve the accuracy of object detection by this model.

#### 3.1.2. Attention Mechanism

**Figure 2:**
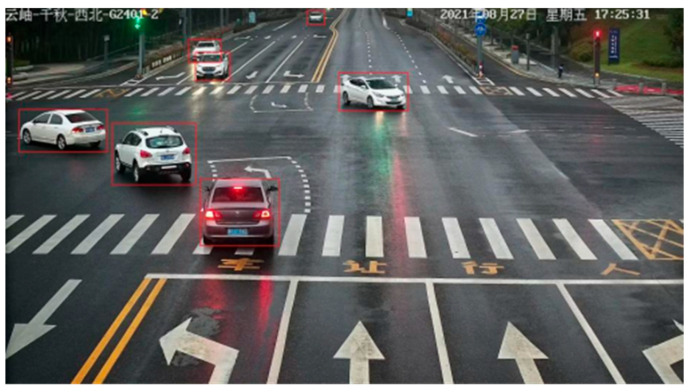
Vehicle detection diagram in data set.

In the object detection task, each part in any picture has a different importance. As shown in Figure 2, the red square in the picture is the visualized result of running the detection model to detect the vehicle. what we need is vehicle-related information to realize the vehicle detection task, so we want to pay more attention to the vehicle-related part. Each part of an image is assigned a weight equal to the amount of attention people pay to each part of the image. In this way, the weight can simulate the different focus of people’s attention when they see the picture, that is, to achieve an attention mechanism. We can use the attention mechanism to improve the model’s attention to traffic targets or small targets, so as to improve the detection ability of the object detection model.

**Figure 3:**
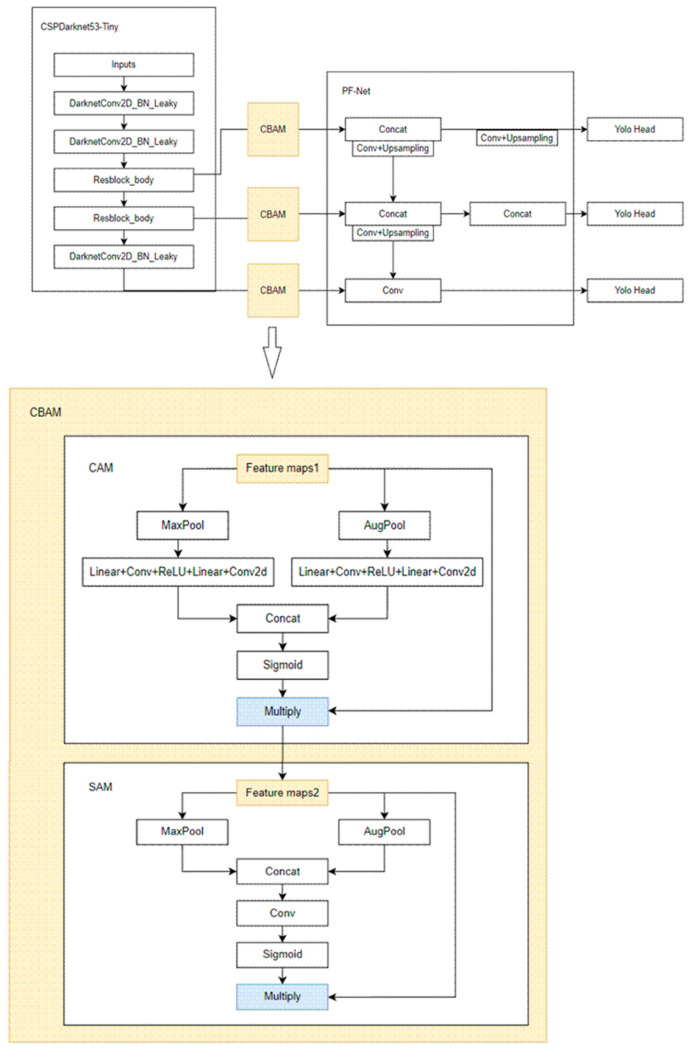
Structure diagram of PF-YOLOv4 tiny-CBAM.

In our model, we use three CBAM structures to improve the model’s focus on important targets and obtain the improved PF-YOLOv4 tiny-CBAM based on YOLOv4 tiny. CBAM is a lightweight structure that can usually be added in any layer of convolution. Using an CBAM attention module in object detection tasks can make the model suppress invalid information areas and pay more attention to areas containing key information in the image. PF-YOLOv4 tiny-CBAM adds a CBAM module after the last convolutional layer of the backbone network, between the first residual module and the PF-Net connection and between the second residual module and the PF-Net connection. Since the last convolutional layer corresponds to the largest target scale and has a large number of channels, it is easy to mix in invalid information, so it is necessary to use the attention mechanism to make the model focus on the feature graph containing effective information. After the residual module, a CBAM module is added because the feature maps corresponding to these two layers are of large scale, and an attention mechanism is needed to make the model pay more attention to the features of the target region. At the same time, adding CBAM modules to these layers does not affect the backbone network, so the weight of the original YOLOv4 tiny can be used for initial training, which makes the model convergence easier, and the model with better precision performance can be obtained. See Figure 3 for the network structure diagram of PF-YOLOv4 tiny-CBAM.

For each CBAM module in Figure 3, the feature figure $F\in R^{C\times H\times W}$ output from different feature layers of the backbone network is taken as input. After CAM processing, the feature figure $F_{1}$ is obtained, and after SAM processing, the final feature figure is $F_{2}$ taken as output. $M_{CAM}\in R^{C\times 1\times 1}$ is a channel attention diagram generated by the channel attention mechanism, and $M_{SAM}\in R^{1\times H\times W}$ is a spatial attention diagram generated by the spatial attention mechanism.

(1) $$F_{1}=M_{CAM}\left(F\right)⊗F$$

(2) $$F_{2}=M_{SAM}\left(F_{1}\right)⊗F_{1}$$

In a CAM module, the feature maps of the backbone network are averaged and maximized, then a shared MLPS and a series of activation operations are used to obtain the channel attention diagram M_CAM_, in which MLP composed of Liner + Conv, etc. are shared parameters. The calculation process of CAM is shown in Formula (3).

(3) $$M_{CAM}\left(F\right)=sigmoid(MLP\left(AvgPool\left(F\right)\right)+MLP(MaxPool\left(F\right)))$$

In a SAM module, the spatial attention diagram $M_{SAM}$ is obtained after averaging and maximum pooling and splicing and then after convolution and activation. The calculation process of SAM is shown in Formula (4).

(4) $$M_{SAM}\left(F_{1}\right)=sigmoid(Conv([AvgPool\left(F_{1}\right);MaxPool(F_{1})]))$$

### 3.2. Data Processing

#### 3.2.1. Data Enhancement

We used the data enhancement method to balance the number of small and medium-sized targets in the data set from the perspective of amplifying small target features. Instead of just following the three methods of enhancement used in the paper that proposed the Copy-Paste method, we made enhancements by selecting a small target in a single image and then copying and pasting several times at random locations in the image. Select multiple small targets in a single image and copy and paste them anywhere in the image. Select all the small targets in a single image and copy and paste them multiple times at any location in that image. We chose to select a number of small targets from the whole data set as the material library of small targets, then select a number of pictures in the data set as the background library and paste random positions on the pictures in the background library by using the targets in the randomly selected material library.

**Figure 4:**
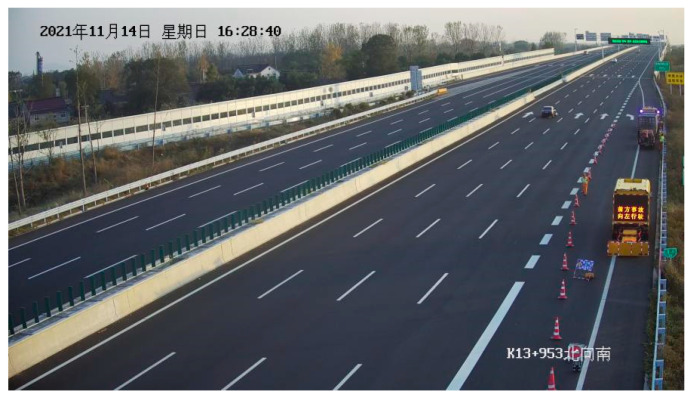
Example of a reflective cone from a camera perspective.

Taking the reflective cone and throwing objects of small target objects in the data set as an example, the data of the reflective cone and throwing objects are generally less, and for the camera perspective, the reflective cone belongs to the small-scale target, as shown in Figure 4.

**Figure 5:**
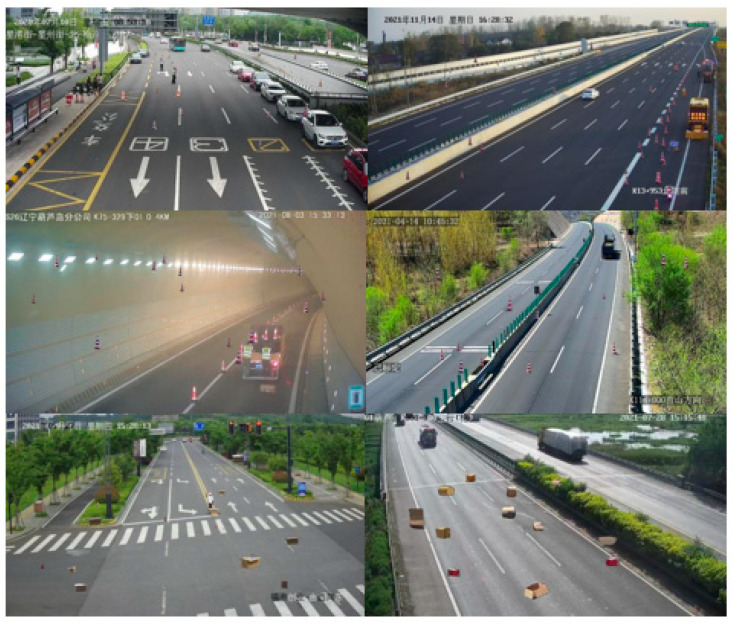
An example diagram of a reflective cone and sprinkles enhanced with Copy-Paste.

However, in real life, it is not easy to collect the data of reflective cones and throwing objects on the pavement. One reason is because there are fewer reflective cones and throwing objects, only in the occurrence of accidents, maintenance, and other situations to be collected. Second, due to road safety and other issues, it is impossible to carry out more artificial creation, such as the placement of reflective cones on the road surface and throwing objects. Generally speaking, the data of reflective cones and throwing objects are suitable for amplification through data enhancement. In this paper, the Copy-Paste method mentioned above is used to enhance the data of reflective cones and throwing objects. The enhanced effect diagram is shown in Figure 5. The enhanced data set contains more target numbers of reflective cones and throwing objects, which can effectively improve the detection ability of the model for such targets.

#### 3.2.2. Prior Bounding Box Clustering

In our method, the K-means algorithm is used to cluster prior bounding boxes, but the calculation method of the distance between the two targets is modified. Euclidean distance is no longer used, but IoU, which is more consistent with the target box, is used for definition. Using Euclidean distance to measure the distance between each target and clustering center, the measurement errors may be related to the size of bounding boxes, and large bounding boxes usually have more errors than small bounding boxes. Therefore, for the target frame, IOU is more appropriate for distance measurement, assuming *anchor* = (*w*_a_, *h*_a_), *box* = (*w*_b_,*h*_b_), where w represents the width of anchor and h represents the height of anchor. See Formulas (5) and (6) for the specific calculation of the intersection ratio of two anchors. In calculation, it is assumed that the center points of all target frames coincide with each other, and only the width and height of the target frames are needed, which can further simplify the calculation. Our complete clustering procedure is shown in Algorithm 1.

(5) $$d\left(box,anchor\right)=1-IOU\left(box,anchor\right)$$

(6) $$d\left(box,anchor\right)=1-\frac{\min\left(w_{a},w_{b}\right)\times \min\left(h_{a},h_{b}\right)}{w_{a}h_{a}+w_{b}h_{b}-\min\left(w_{a},w_{b}\right)\times \min\left(h_{a},h_{b}\right)}$$

**Algorithm 1.** K-means clustering process**Input:** image1, …, imageN annotated data **Output:** 9 anchors of different widths and heights 1: Initially, 9 anchors given in COCO data set are selected as the clustering center, and the number of clustering centers is set as k = 9. 2: Calculate the distance between each target a in the data set and each cluster center b:         $\begin{array}{c} d=1-\frac{\min\left(w_{a},w_{b}\right)\times \min\left(h_{a},h_{b}\right)}{w_{a}h_{a}+w_{b}h_{b}-\min\left(w_{a},w_{b}\right)\times \min\left(h_{a},h_{b}\right)} \end{array}$ 3: The class is divided according to the value of d. 4: **repeat** 5: **until** (iters≥150)

The clustering results are as follows: 10, 10, 24, 16, 21, 40, 52, 28, 48, 84, 114, 48, 120, 117, 280, 145, 519, 278.

## 4. Experiment

### 4.1. Introduction to Data Sets

The object detection algorithm in this paper was applied to the traffic camera perspective to provide real-time target categories and positions required in camera images for the subsequent determination of traffic congestion, traffic violations, and other tasks. The data set was based on traffic images from the public data set COCO and VOC data sets and mainly included road monitoring data from several cities in China, including scenes such as highways and intersections, which were amplified by camera images.

In order to make the model provide upstream detection results for more tasks in the future, the data set contained more categories, a total of 31 targets. Before data enhancement, it mainly contained about 200,000 data points, and the resolution of most images was 1920 × 1080, about 32 G, which met the basic data requirements of object detection model training. During the experiment, we mainly focused on the categories of person, car, reflective cone, and sprinkles, which contained many small-scale objects. The model training size was selected to conform to 608 × 320 of 1920 × 1080.

The data categories included: bicycle, bus, car, bicycle, motorcycle, pedestrian, truck, tricycle, person riding non-motorized vehicle, rearview mirror, car light barrier, car windshields, car pendant, vehicle annual inspection sign, pass check, seat belt, license plate, car light, roof window, car luggage rack, practice sign, wheel, interior item, painted vehicle license plate, vehicle hazardous chemical sign, traffic sign, reflective cone, triangle sign, litter, stroller, and handcart. These categories were set based on some traffic-related events. They were placed in the same model to reduce the consumption of computing resources caused by calling multiple models in practical applications. The ratio of the training data set, validation data set, and test data set was approximately 7:2:1, and each scene captured by each camera was divided according to this ratio to form the final training data set, validation data set, and test data set.

### 4.2. Model Structure Comparison Experiment

In this paper, a new multi-scale feature fusion method, PF-Net, is proposed, and the attention module CBAM is added. The experimental results of these two structures will be compared in the following sections.

It can be seen from Table 1 that the modified PF-Net structure can effectively improve the detection accuracy of the model for categories of focus, such as person, car, etc. At the same time, the model with the addition of the attention-mechanism CBAM module, compared with both YOLOv4 tiny and PF-YOLOv4 tiny, further improves the detection effect of these and can reach up to three to four percentage points of improvement in individual categories.

**Figure 6:**
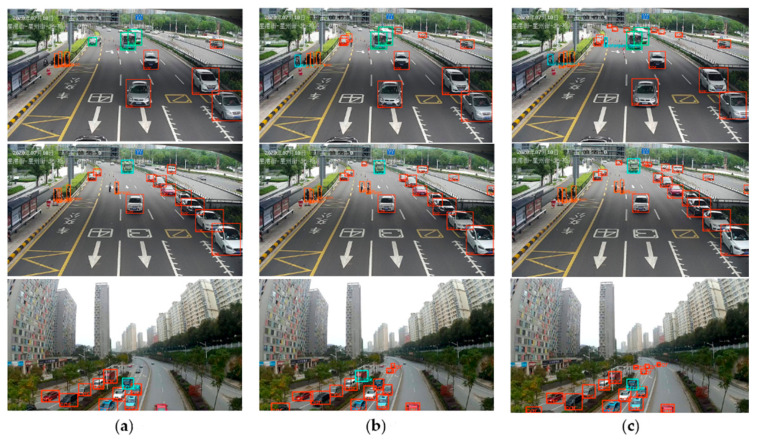
Comparison of detection results of various algorithms. (**a**) YOLOv4 tiny test results; (**b**) PF-YOLOv4 tiny test results; (**c**) PF-YOLOv4 tiny-CBAM test results.

A 0.2 confidence threshold for display was selected, and images that did not contain scenes in the training set were selected for the test of YOLOv4 tiny, PF-YOLOv4 tiny, PF-YOLOv4 tiny-CBAM models, and representative test diagrams were selected for analysis, as shown in Figure 6. In Figure 6, the boxes in the figure represent the detection results of running different detection models, and the boxes of different colors represent the different categories detected, such as the red detection box corresponding to car. And the bottom left corner of the box in the figure displays the corresponding detection category results.

It can be seen from the detection diagram that the detection effect of the improved PF-YOLOv4 tiny and PF-YOLOv4 tiny-CBAM model is better than that of the original YOLOv4 tiny model. The improved model can detect some small targets better than the original model, such as people, distant cars, and reflective cones, which proves the effectiveness of the improved model. Parts of the false detection and missing detection still need to be further optimized from the aspect of data, which will be elaborated and tested in Section 4.3.

The experiments in Table 2 prove that adding an additional anchor head and increasing six anchors to nine anchors allow the model to better adapt to multiple anchors at multiple scales, and the model’s ability to examine multiple categories in the data set will be enhanced to varying degrees. Using an improved PFNet structure instead of the original FPN in the model enables the model to expand the size span of the detected targets, while using a repeated feature fusion structure for small and medium-sized targets to ensure improved accuracy for these relatively small targets. This improvement can increase mAP by 2.01%. The added channel attention module increases mAP by 1.35 percentage points over the improved PF-YOLOv4 tiny; compared to YOLOv4 tiny, it has increased by 4.03%. Taking the reflector cone as an example, the final improved model PF-YOLOv4 tiny CBAM can increase by 1.69 percentage points and also ensures that the accuracy of most categories is improved relative to the PF-YOLOv4 tiny. PF-YOLOv4 tiny-CBAM is equivalent to further improving the detection performance on the basis of guaranteeing the detection capability of PF-YOLOv4 tiny.

According to the real-time experiment (Table 2), although PF-YOLOv4 tiny and PF-YOLOv4 tiny-CBAM with modified structure detect fewer images per second than YOLOv4 tiny, they still have the characteristics of real-time and can be applied to an intelligent transportation system. The improvement is a kind of precision improvement, at the expense of a small amount of real-time performance.

### 4.3. Comparative Experiment Based on Data

The effectiveness verification experiments of Copy-Paste mainly show the effects of reflecting cones and throwing objects.

As can be seen from Table 3, the model after data enhancement has stronger detection ability for several categories of enhancement and can achieve a maximum accuracy improvement of three to four percentage points. Compared with PF-YOLOv4 tiny, the improved model with the CBAM module added at the same time has higher detection accuracy. The attention mechanism of PF-YOLOv4 tiny-CBAM plays a role, making the model pay more attention to the target in the image, so as to obtain a better detection effect.

**Figure 7:**
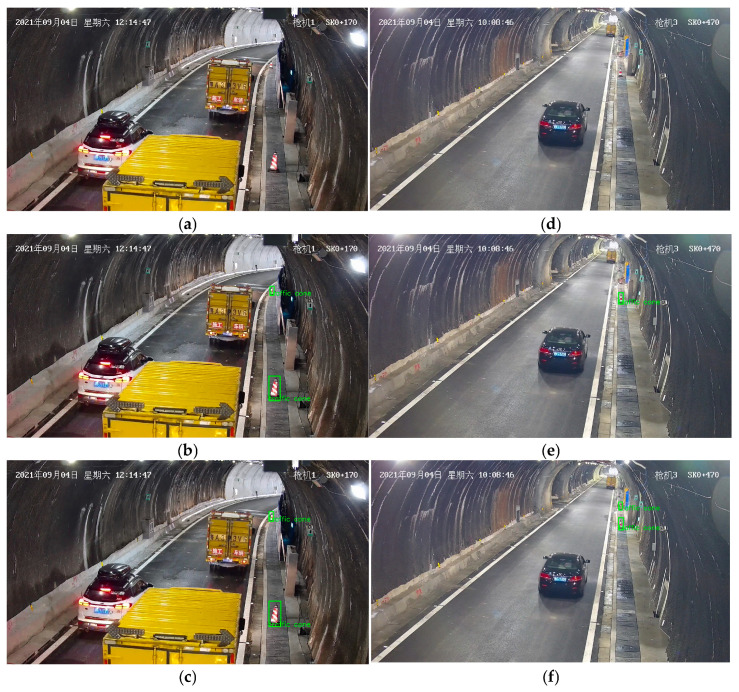
Reflection cone detection effect. (**a**) Example 1: using PF-YOLOv4-tiny-CBAM model without data enhancement; (**b**) example 1: Using PF-YOLOv4-tiny model with data enhancement; (**c**) example 1: using PF-YOLOv4-tiny-CBAM model with data enhancement; (**d**) example 2: using PF-YOLOv4-tiny-CBAM model without data enhancement; (**e**) example 2: using PF-YOLOv4-tiny model with data enhancement; (**f**) example 2: using PF-YOLOv4-tiny-CBAM model with data enhancement.

Take reflection cone detection as an example, select scene pictures not included in the training set for testing, and the representative test results are shown in Figure 7. Figure 7a uses PF-YOLOv4-tiny-CBAM model without data enhancement. It can be seen that although the structure has been modified and the overall detection accuracy has been improved, the amount of data for the reflection cone is small. Without sufficient training data, it still cannot be detected. For this category, the generalization of the model cannot achieve good results. Figure 7b,c show the detection effect of PF-YOLOv4-tiny and PF-YOLOv4-tiny-CBAM, which are trained with data enhanced using Copy-Paste. In Figure 7d–f, the same is true. It can be seen that the model with enhanced data has better detection ability for reflective cones, and the PF-YOLOv4-tiny-CBAM with enhanced data has better generalization.

In the unamplified data set, there was a single scene of reflective cones and sprinkles, a small number of targets for this category, and poor generalization. As shown in Figure 7 and Table 3 above, the generalization ability of the model trained with the data set enhanced by data has been enhanced for the reflective cone. Through data enhancement, the model’s learning of the features of this category has been improved, and thus its detection and generalization ability for this category has been improved.

After K-means was used for clustering, the improved version of YOLOv4 tiny model was used, and the detection effects of various categories are shown in Table 4.

It can be found through the experiment that both the improved PF-YOLOv4 tiny and PF-YOLOv4 tiny-CBAM can improve the AP value of most categories after K-means clustering for anchor. It is worth noting that the improvement span of detection accuracy of categories such as car and person is higher than that of some other categories, which increases by about 3%. This may be because the categories such as car have a large number of targets. K-means clustering can have a greater influence on the clustering center, and anchors more suitable for these categories can be obtained. It is further explained that selecting the prior bounding box ratio of the appropriate data set is helpful to improve the detection ability of the model. The final improved PF-YOLOv4 tiny CBAM + Copy-Paste + K-means model increased mAP by 4.9% compared to the original YOLOv4 tiny.

## 5. Conclusions

In this paper, an improved model based on YOLOv4 tiny is proposed to address the issue of small pedestrian targets in some vehicles in intelligent transportation scenarios. Based on the FPN structure, the number of detection heads has been increased, and a top-down feature fusion path has been added for small and medium-sized targets. At the same time, a CBAM module has been added to assist in enhancing the model’s ability to detect small targets and ensuring its real-time performance. Tested on a 260,000 custom traffic data set containing some public traffic images, this improvement improved the model’s mAP by 4.03%, and the detection accuracy for small targets was also correspondingly improved. To address the issue of imbalanced data and features in custom traffic data sets containing public VOC and COCO partial traffic images, an improved Copy-Paste is used to enhance the features of some categories, ensuring that the AP values of the corresponding categories have at least one point of improvement. Using K-means with improved distance measurement to solve the mismatch problem between the data set and prior bounding boxes, some categories can achieve a three-percentage-point improvement.

## Figures and Tables

**Table 1:** Evaluation results on test data of custom traffic data set (AP/%).

	Class	Person	Car	Reflector Cone	Throwing Objects
Model					
YOLOv4 tiny		69.40%	82.28%	66.92%	90.47%
PF-YOLOv4 tiny		70.59%	84.27%	67.61%	91.49%
PF-YOLOv4 tiny-CBAM		73.79%	86.07%	68.61%	91.09%

**Table 2:** Comparison of mAP and real-time results of the model.

	Item	mAP	FPS
Model			
YOLOv4 tiny		60.68%	93
PF-YOLOv4 tiny		62.69%	87
PF-YOLOv4 tiny-CBAM		64.04%	81

**Table 3:** Detection effect of the corresponding category after data set amplification (AP/%).

	Class	Reflector Cone	Throwing Objects
Model			
YOLOv4 tiny		66.92%	90.47%
PF-YOLOv4 tiny		67.61%	91.49%
PF-YOLOv4 tiny-CBAM		68.61%	91.09%
PF-YOLOv4 tiny + Copy-Paste		68.41%	92.54%
PF-YOLOv4 tiny-CBAM + Copy-Paste		69.32%	91.98%

**Table 4:** Detection effect of corresponding categories after anchor clustering by K-means (AP/%).

	Class	Person	Car	Reflector Cone	Throwing Objects
Model					
YOLOv4 tiny		69.40%	82.28%	66.92%	90.47%
PF-YOLOv4 tiny		70.59%	84.27%	67.61%	91.49%
PF-YOLOv4 tiny-CBAM		73.79%	86.07%	68.61%	91.09%
PF-YOLOv4 tiny + Copy-Paste + K-means		73.79%	86.73%	69.41%	92.14%
PF-YOLOv4 tiny-CBAM +Copy-Paste + K-means		76.99%	89.05%	70.32%	91.58%

## Data Availability

The dataset involved in this study, though of immense academic value, poses a moral dilemma for us in terms of its public disclosure. This stems primarily from the fact that the dataset contains a vast amount of personal sensitive information and implicates the privacy rights of specific groups. Given the sensitivity and gravity of these issues, we are confronted with the challenging task of balancing the pursuit of academic advancement with the protection of individual privacy. Furthermore, it is crucial to note that the dataset for this research is sourced from collaborating enterprises with whom we have established agreements and commitments. These agreements explicitly restrict our authority to disclose the dataset without prior consent. Thus, the legal and contractual obligations we have undertaken further compound the inability to make the dataset publicly available.

## References

[1] Kisantal M., Wojna Z., Murawski J., Naruniec J., Cho K. (2019). Augmentation for small object detection. arXiv.

[2] Liu Y., Sun P., Wergeles N., Shang Y. (2021). A survey and performance evaluation of deep learning methods for small object detection. Expert Syst. Appl..

[3] Lin T.Y., Dollár P., Girshick R., He K., Hariharan B., Belongie S. Feature Pyramid Networks for Object Detection. Proceedings of the IEEE Conference on Computer Vision and Pattern Recognition.

[4] Bochkovskiy A., Wang C.Y., Liao H.Y.M. (2020). Yolov4: Optimal speed and accuracy of object detection. arXiv.

[5] Cheng G., Yuan X., Yao X., Yan K., Zeng Q., Xie X., Han J. (2022). Towards large-scale small object detection: Survey and benchmarks. arXiv.

[6] Macqueen J. Some Methods for Classification and Analysis of Multivariate Observations. Proceedings of the 5th Berkeley Symposium on Mathematical Statistics and Probability.

[7] Girshick R., Donahue J., Darrell T., Malik J. Rich Feature Hierarchies for Accurate Object Detection and Semantic Segmentation. Proceedings of the IEEE Conference on Computer Vision and Pattern Recognition.

[8] He K., Zhang X., Ren S., Sun J. (2015). Spatial pyramid pooling in deep convolutional networks for visual recognition. IEEE Trans. Pattern Anal. Mach. Intell..

[9] Girshick R. Fast r-cnn. Proceedings of the IEEE International Conference on Computer Vision.

[10] Ren S., He K., Girshick R., Sun J. (2015). Faster r-cnn: Towards real-time object detection with region proposal networks. Adv. Neural Inf. Process. Syst..

[11] Liu W., Anguelov D., Erhan D., Szegedy C., Reed S., Fu C.Y., Berg A.C. (2016). Ssd: Single shot multibox detector. Proceedings of the ECCV 2016: 14th European Conference on Computer Vision.

[12] Redmon J., Farhadi A. (2018). Yolov3: An incremental improvement. arXiv.

[13] Duan K., Bai S., Xie L., Qi H., Huang Q., Tian Q. Centernet: Keypoint Triplets for Object Detection. Proceedings of the IEEE/CVF International Conference on Computer Vision.

[14] Zhu C., He Y., Savvides M. Feature Selective Anchor-Free Module for Single-Shot Object Detection. Proceedings of the IEEE/CVF Conference on Computer Vision and Pattern Recognition.

[15] Chen Y., Zhang P., Li Z., Li Y., Zhang X., Qi L., Sun J., Jia J. (2020). Feedback-driven data provider for object detection. arXiv.

[16] Ghiasi G., Cui Y., Srinivas A., Qian R., Lin T.Y., Cubuk E.D., Le Q.V., Zoph B. Simple Copy-Paste is a Strong Data Augmentation Method for Instance Segmentation. Proceedings of the IEEE/CVF Conference on Computer Vision and Pattern Recognition.

[17] Zhang S., Zhu X., Lei Z., Shi H., Wang X., Li S.Z. Faceboxes: A CPU real-time face detector with high accuracy. Proceedings of the 2017 IEEE International Joint Conference on Biometrics (IJCB).

[18] Zhang S., Zhu X., Lei Z., Shi H., Wang X., Li S.Z. S3fd: Single Shot Scale-INVARIANT face Detector. Proceedings of the IEEE International Conference on Computer Vision.

[19] Xu C., Wang J., Yang W., Yu L. Dot distance for tiny object detection in aerial images. Proceedings of the IEEE/CVF Conference on Computer Vision and Pattern Recognition.

[20] Shannon C.E. (1948). A mathematical theory of communication. Bell Syst. Tech. J..

[21] Lin T.Y., Goyal P., Girshick R., He K., Dollár P. Focal Loss for Dense Object Detection. Proceedings of the IEEE International Conference on Computer Vision.

[22] Tan M., Pang R., Le Q.V. Efficientdet: Scalable and Efficient Object Detection. Proceedings of the IEEE/CVF Conference on Computer Vision and Pattern Recognition.

[23] Liu S., Huang D., Wang Y. (2019). Learning spatial fusion for single-shot object detection. arXiv.

[24] Qiao S., Chen L.C., Yuille A. Detectors: Detecting Objects with Recursive Feature Pyramid and Switchable Atrous Convolution. Proceedings of the IEEE/CVF Conference on Computer Vision and Pattern Recognition.

[25] Ge L., Dan D., Li H. (2020). An accurate and robust monitoring method of full-bridge traffic load distribution based on YOLO-v3 machine vision. Struct. Control. Health Monit..

[26] Ge L., Dan D., Koo K.Y., Chen Y. (2023). An Improved System for Long-Term Monitoring of Full-Bridge Traffic Load Distribution on Long-Span Bridges. Structures.

[27] Lin C.J., Jhang J.Y. (2022). Intelligent traffic-monitoring system based on YOLO and convolutional fuzzy neural networks. IEEE Access.

[28] Al Mudawi N., Qureshi A.M., Abdelhaq M., Alshahrani A., Alazeb A., Alonazi M., Algarni A. (2023). Vehicle detection and classification via YOLOv8 and deep belief network over aerial image sequences. Sustainability.

